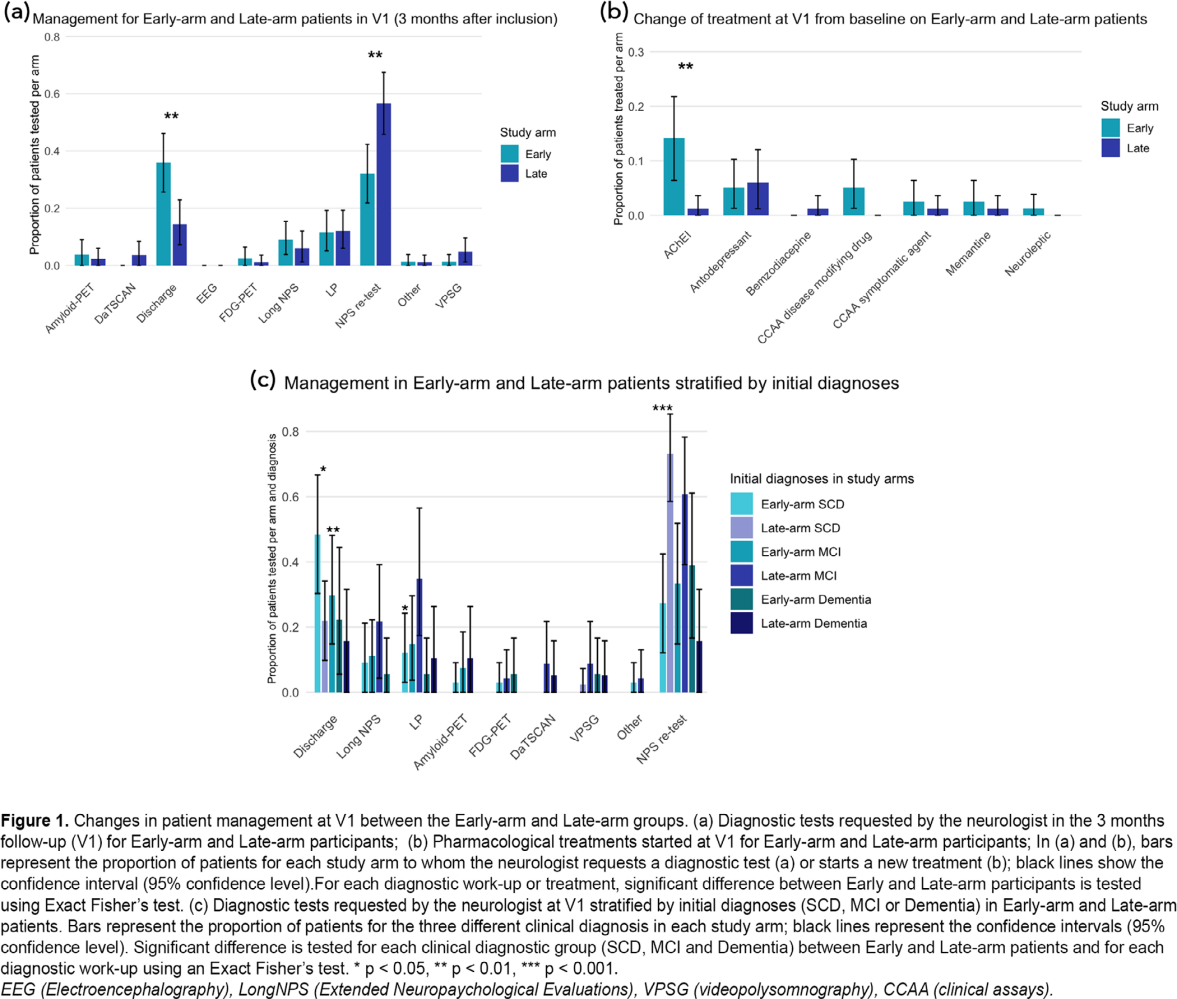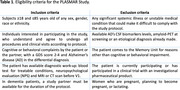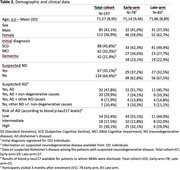# Early Disclosure of Blood‐Based Biomarkers Optimizes Alzheimer's Disease Management and Treatment Initiation: the PLASMAR study

**DOI:** 10.1002/alz70856_106918

**Published:** 2026-01-08

**Authors:** Isabel Estragués‐Gázquez, José Contador, Leidy Dayana Martinez, Aida Fernández‐Lebrero, Gianmarco Iaccarino, Greta Garcia‐Escobar, Rosa Maria Manero‐Borràs, Irene Navalpotro‐Gómez, Oriol Grau‐Rivera, Paula Ortiz‐Romero, Marina De Diego‐Osaba, Helena Blasco‐Forniés, Esther Jiménez‐Moyano, Javier Torres‐Torronteras, Juan José Hernández Sánchez, Anna Padrós, Pablo Villoslada, Marta del Campo, Albert Puig‐Pijoan, Marc Suarez‐Calvet

**Affiliations:** ^1^ Barcelonaβeta Brain Research Center (BBRC), Pasqual Maragall Foundation, Barcelona, Spain; ^2^ Hospital del Mar Research Institute (IMIM), Barcelona, Spain; ^3^ Cognitive Decline Unit, Neurology service, Hospital del Mar, Barcelona, barcelona, Spain; ^4^ Cognitive Decline Unit, Department of Neurology, Hospital Del Mar, Barcelona, Spain; ^5^ BarcelonaBeta Brain Research center, Barcelona, Barcelona, Spain; ^6^ Hospital del Mar Research institute, Barcelona, Barcelona, Spain; ^7^ Hospital del Mar Medical Research Institute (IMIM), Barcelona, Spain; ^8^ BarcelonaBeta Brain Research Center, Barcelona, Barcelona, Spain; ^9^ Servei de Neurologia, Hospital del Mar, Barcelona, Spain; ^10^ Centro de Investigación Biomédica en Red de Fragilidad y Envejecimiento Saludable (CIBERFES), Instituto de Salud Carlos III, Barcelona, Spain; ^11^ Barcelonaβeta Brain Research Center (BBRC), Barcelona, Spain; ^12^ Laboratori de Referència de Catalunya, Barcelona, Barcelona, Spain; ^13^ Hospital del Mar Research Institute, Barcelona, Barcelona, Spain; ^14^ Pompeu Fabra University, Barcelona, Catalonia, Spain; ^15^ Neurology service, Hospital del Mar, Barcelona, Barcelona, Spain; ^16^ Departamento de Ciencias Farmacéuticas y de la Salud, Facultad de Farmacia, Universidad San Pablo‐CEU, CEU Universities, Urbanización Montepríncipe, Spain; ^17^ Centro de Investigación Biomédica en Red de Fragilidad y Envejecimiento Saludable (CIBERFES), Instituto de Salud Carlos III, Madrid, Spain; ^18^ Cognitive Decline and Movement Disorders Unit, Neurology Department, Hospital del Mar, Barcelona, Spain

## Abstract

**Background:**

Blood‐based biomarkers (BBMs) offer a cost‐effective, non‐invasive approach for detecting Alzheimer's disease (AD) pathology, but their impact on improving diagnostic certainty and patient management remains unclear. The PLASMAR study is a prospective, single‐center, blinded, randomized controlled trial assessing the effect of early versus delayed BBM adoption on clinical practice in a public hospital memory clinic. This abstract presents an interim analysis on changes in patient management.

**Method:**

From February to October 2024, 224 patients with cognitive or behavioral complaints (GDS 2–4) referred to the memory clinic at Hospital del Mar (Barcelona, Spain) were prospectively enrolled. Eligibility criteria are detailed in Table 1. At baseline, blood samples were collected for BBMs, and neurologists assessed diagnostic confidence based on routine clinical evaluation without BBM results. Participants were randomized to the Early (BBM results disclosed at 3 months) or Late (disclosed at 9 months) arms. Patient management changes were compared between arms. Plasma *p*‐tau217 levels, classified by validated cutoffs, stratified patients into low, intermediate, or high risk for AD pathology.

**Result:**

Of 224 enrolled patients, 27 were lost to follow‐up, leaving 197 participants (mean age 71.6 ± 8.9 years; 56.9% female). There were no demographic differences between study arms. Initial diagnoses included SCD (44.7%), MCI (31.4%), or dementia (21.3%), with AD suspected in 30%. By December 2024, 114 patients (87 Early, 27 Late) had BBM results disclosed. AD risk was classified as low (52.4%), intermediate (17.5%), or high (30%). At 3‐months follow‐up, Early‐arm patients had higher discharge rates and initiated AchEIs sooner (*p* <0.01), while Late‐arm patients more frequently received longitudinal cognitive monitoring (*p* <0.01). Among SCD patients, Early‐arm participants had higher discharge rates and underwent more lumbar punctures for CSF biomarkers according to their AD pathology risk (*p* <0.05), while Late‐arm patients were more likely to undergo cognitive monitoring (*p* <0.001). Among MCI patients, Early‐arm participants had higher discharge rates (*p* <0.01). Similar trends were observed in the low‐risk AD group (*p* <0.05).

**Conclusion:**

This interim analysis demonstrates that early disclosure of BBM results influences patient management and accelerates the initiation of specific AD treatments, also reducing the need for follow‐up of cognitive assessments.